# Rupture of the Spleen

**Published:** 1826-07

**Authors:** 


					Rupture of the Spleen.
M. Bailly, who has written a very interesting
,v?rlv I""*'u "J (ne opiecil. ivi. Joainy, who uas wnueu u very mieresuug
of the>c,t^le *ntermittent remittent fevers of Italy, and particularly those
c?m,no *mSna ^om:i> informs us that rupture of the spleen is a very
. UCCd t 1 ? O S 1V1UI uuu l|,a SUUkWlk u?uivv... v.w 11 ?m
c'tculat" ? -1 u' putrid jelly. These effects of the violent orgasms of th
^?es not1?^CVC1' 'lc attributes very properly to sanguineous congestion, and
u?k upon them as at all the causes but the consequences of the fever.
L

				

